# Dirichlet process mixture models for single-cell RNA-seq clustering

**DOI:** 10.1242/bio.059001

**Published:** 2022-04-04

**Authors:** Nigatu A. Adossa, Kalle T. Rytkönen, Laura L. Elo

**Affiliations:** 1 Turku Bioscience Centre, University of Turku and Åbo Akademi University, FI-20520, Turku, Finland; 2 Institute of Biomedicine, Research Centre for Integrative Physiology and Pharmacology, University of Turku, FI-20014, Finland; 3 Institute of Biomedicine, University of Turku, FI-20014, Finland

**Keywords:** Clustering, Hierarchical Dirichlet process (HDP), Latent Dirichlet allocation (LDA), ScRNA-seq

## Abstract

Clustering of cells based on gene expression is one of the major steps in single-cell RNA-sequencing (scRNA-seq) data analysis. One key challenge in cluster analysis is the unknown number of clusters and, for this issue, there is still no comprehensive solution. To enhance the process of defining meaningful cluster resolution, we compare Bayesian latent Dirichlet allocation (LDA) method to its non-parametric counterpart, hierarchical Dirichlet process (HDP) in the context of clustering scRNA-seq data. A potential main advantage of HDP is that it does not require the number of clusters as an input parameter from the user. While LDA has been used in single-cell data analysis, it has not been compared in detail with HDP. Here, we compare the cell clustering performance of LDA and HDP using four scRNA-seq datasets (immune cells, kidney, pancreas and decidua/placenta), with a specific focus on cluster numbers. Using both intrinsic (DB-index) and extrinsic (ARI) cluster quality measures, we show that the performance of LDA and HDP is dataset dependent. We describe a case where HDP produced a more appropriate clustering compared to the best performer from a series of LDA clusterings with different numbers of clusters. However, we also observed cases where the best performing LDA cluster numbers appropriately capture the main biological features while HDP tended to inflate the number of clusters. Overall, our study highlights the importance of carefully assessing the number of clusters when analyzing scRNA-seq data.

## Abstract (precis)

**Summary:** Dirichlet mixture models (LDA and HDP) are applied for clustering cells in scRNA-Seq data. Here we made a comprehensive comparison of LDA and HDP model-based clustering for scRNA-seq data.

## INTRODUCTION

Recent advances in single-cell sequencing have enabled increased resolution of biological and medical studies of cellular functions. Single-cell RNA-sequencing (scRNA-seq) is widely used to study cellular heterogeneity in cancer, developmental biology, immunology and neurology (Tang et al., 2019). Clustering of cells based on their gene expression profiles is one of the major steps in scRNA-seq data analysis. For instance, the computational analysis of scRNA-seq data for cell-type identification has mainly relied on unsupervised clustering methods (Qi et al., 2019), such as distance-based cluster optimization, density-based clustering, or graph-based clustering methods (Petegrosso et al., 2019).

Single-cell clustering methods are mainly based on optimization of the pairwise distance between cells (Petegrosso et al., 2019; Qi et al., 2019), which is a challenging task due to the high dimensionality of the data (Remesh and Pattabiraman, 2017). The choice of the distance metric also affects the clustering result (Singh et al., 2013). Bayesian clustering, which uses sampling-based inference methods for clustering, can be used to address these challenges. Unlike traditional distance optimization-based techniques, the Bayesian approach uses soft cluster assignments, in which the data points are assigned to each cluster according to their probability of uncertainty, allowing a mixed cluster membership. Moreover, sampling-based Bayesian clustering methods avoid distance calculation, allowing a tractable way of dealing with high dimensional data. One such Bayesian admixture model is latent Dirichlet allocation (LDA) (Blei et al., 2003), which recently has been successfully adopted for clustering of both scRNA-seq (Dey et al., 2017; duVerle et al., 2016; Sun et al., 2018; Wang et al., 2021) and scATAC-seq (Bravo González-Blas et al., 2019) data.

One key challenge in cluster analysis is the choice of cluster resolution. This is inherently linked to one of the great advances of single-cell sequencing, which is the discovery of previously unknown cellular states or even new cell types. There are several clustering methods available for scRNA-seq data analysis with different parameters regulating the cluster resolution. For instance, Seurat 4 (Hao et al., 2021) implements the shared-nearest neighbor (SNN) graph-based clustering on PCA space with modularity optimization and a user-selected parameter regulating the cluster resolution (Butler et al., 2018). Similarly, Monocle 3 (Cao et al., 2019) implements graph-based community detection algorithms with a user-defined input resolution parameter. However, an inappropriate choice of these parameters may impede the discovery of novel cell states or types.

To address these challenges, in this study, we investigate the utility of hierarchical Dirichlet process (HDP) (Teh et al., 2006) for clustering scRNA-seq data as a non-parametric counterpart of LDA. The HDP method has been applied, for example, to correct technical variations for scRNA-seq data (Prabhakaran et al., 2016), to segment gene regulatory networks (Wang and Wang, 2013) and to cluster bulk gene expression data (Wang and Wang, 2013). Here we apply HDP to cluster scRNA-seq data and compare its performance to LDA. We analyze in detail three publicly available scRNA-seq datasets, including artificially mixed human immune cells, and two tissue-specific subsets of kidney and pancreas cells from *Tabula Muris* (Schaum et al., 2018), with high quality cell-type annotations. Additionally, we also test the scalability of the methods with a large dataset from human decidua/placenta (Vento-Tormo et al., 2018). We specifically focus on the clustering resolution necessary to capture the cellular heterogeneity using both intrinsic and extrinsic cluster quality measures.

## RESULTS

To study the performance of LDA and HDP clustering models in identifying the cellular heterogeneity from scRNA-seq data, we applied them to an artificial mixture of human immune cells (Table S1), mouse kidney cells, and mouse pancreas cells (Schaum et al., 2018). For each dataset, the cluster quality was measured first intrinsically using the Davies-Bouldin index (DB-index) and secondly extrinsically using the Adjusted Rand Index (ARI) with the reference clusters from the original publications (see Materials and Methods for details). In addition to DB-index, we also tested the intrinsic cluster quality with Calinski-Harabasz (CH-index) (Calinski and Harabasz, 1974), which overall gave similar results as the DB-index (Fig. S1). Finally, the clustering results of the best two *k* values based on the intrinsic DB-index were visualized using the UMAP plots side by side with the reference cell-type annotations from the original publications. In each dataset, we ran HDP clustering with 20 repetitions and a series of LDA clusterings with an increasing number of clusters *k* from 2 to 20 (20 repetitions each) using the default parameters. The run time for a single analysis on a 48 core Ubuntu 16.04 EC2 cloud instance was ∼2-3 min for LDA in the immune cells (∼1000 cells), pancreas cells (∼2000 cells) and kidney cells (∼3000 cells), whereas the run time for HDP increased from ∼6 min with ∼1000 cells to ∼15 min with ∼2000 cells and ∼28 min with ∼3000 cells (Table S2). LDA and HDP run times for the decidua/placenta (64,000 cells) took 1.35 h and 4 days respectively, and this data was not used for the full comparison between LDA and HDP. The memory usage was similar between LDA and HDP (Table S2).

### LDA and HDP clustering performance in human immune cells

**Fig. 1.**
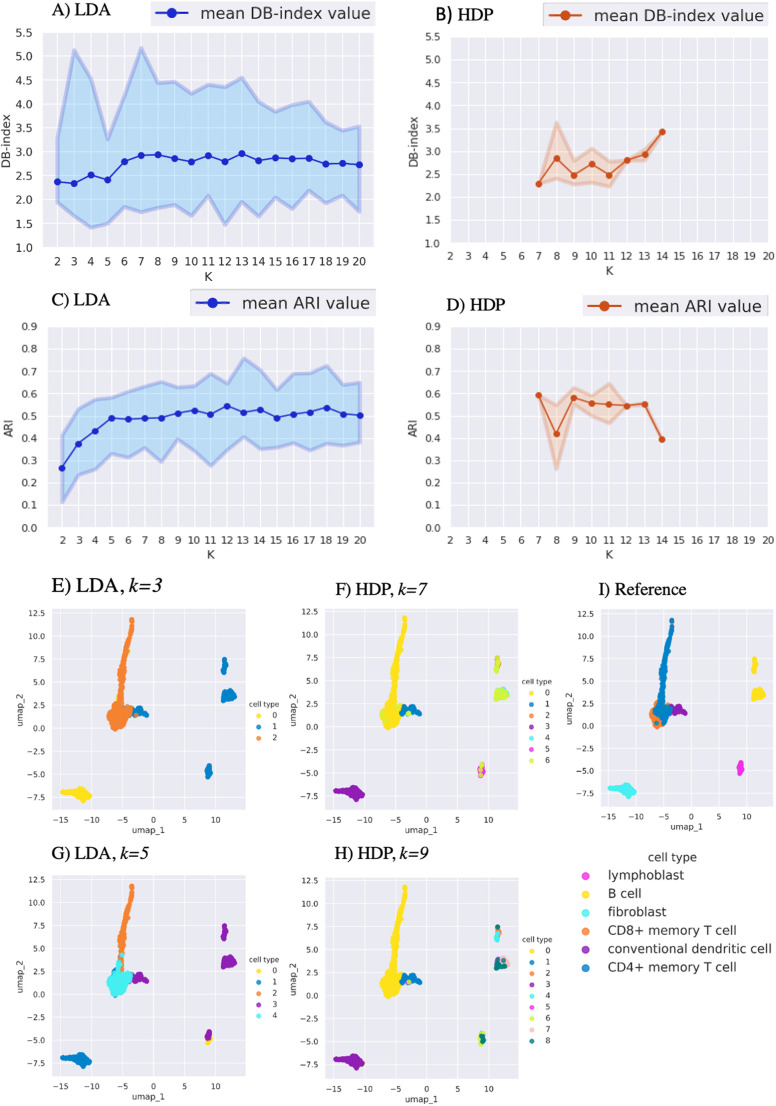
**Comparison of LDA and HDP clustering performance using artificially mixed human immune cell scRNA-seq data.** Intrinsic cluster quality measure defined by DB-index for (A) LDA and (B) HDP clustering. The *x*-axis shows the number of clusters *k*, and the *y*-axis indicates the DB-index values (lower indicates better clustering). Extrinsic cluster quality measure defined by ARI for (C) LDA and (D) HDP clustering. The *x*-axis shows the number of clusters *k*, and the *y*-axis indicates ARI (higher indicates better clustering). For A-D each run was repeated 20 times and the top, middle and bottom lines show the maximum, mean and minimum quality values, respectively. The UMAP plot of LDA clustering with (E) *k*=3 and (G) *k*=5. The UMAP plot of HDP clustering with (F) *k*=7 and (H) *k*=9. (I) The UMAP plot showing the reference clustering with the cell-type annotation from the original publications (Table S1).

In the intrinsic evaluation of the human immune cell data, the two lowest (best) DB-index values with LDA were obtained with *k*=3 (DB=2.3) and *k*=5 (DB=2.4) clusters (Fig. 1A), whereas for HDP those were *k*=7 (DB=2.3) and *k*=9 (DB=2.4) (Fig. 1B).

In the extrinsic cluster evaluation, increasing the LDA cluster number to *k*=5 resulted in an increasingly better quality in terms of ARI, but larger cluster numbers did not affect the quality markedly (Fig. 1C). The mean ARI values for two best DB-index informed HDP clusterings (*k*=7 and *k*=9) had higher ARI values (∼0.6) than those of LDA (<0.5 for *k*=3 and *k*=5) (Fig. 1D). Thus, the extrinsic quality measures were in line with the intrinsic DB-index values, suggesting that – judged by the reference clusters – HDP performed slightly better than LDA in this dataset.

We next visually inspected the best performing clusterings selected by DB-index with UMAP plots by comparing these to the reference cell-type annotations (Fig. 1E-I). HDP with *k*=7 resolved the main reference cell types (Fig. 1F), whereas LDA with *k*=5 did not (Fig. 1G). Specifically, LDA with *k*=5 had one cluster containing B cells, dendritic cells and lymphoblasts together, whereas HDP with *k*=7 or *k*=9 was able to resolve these three cell types to their own clusters. Overall, for this dataset, when comparing with the reference clusters, the DB-index informed HDP was able to predict a biologically more adequate clustering than DB-index informed LDA.

### LDA and HDP clustering performance in mouse kidney cells

**Fig. 2.**
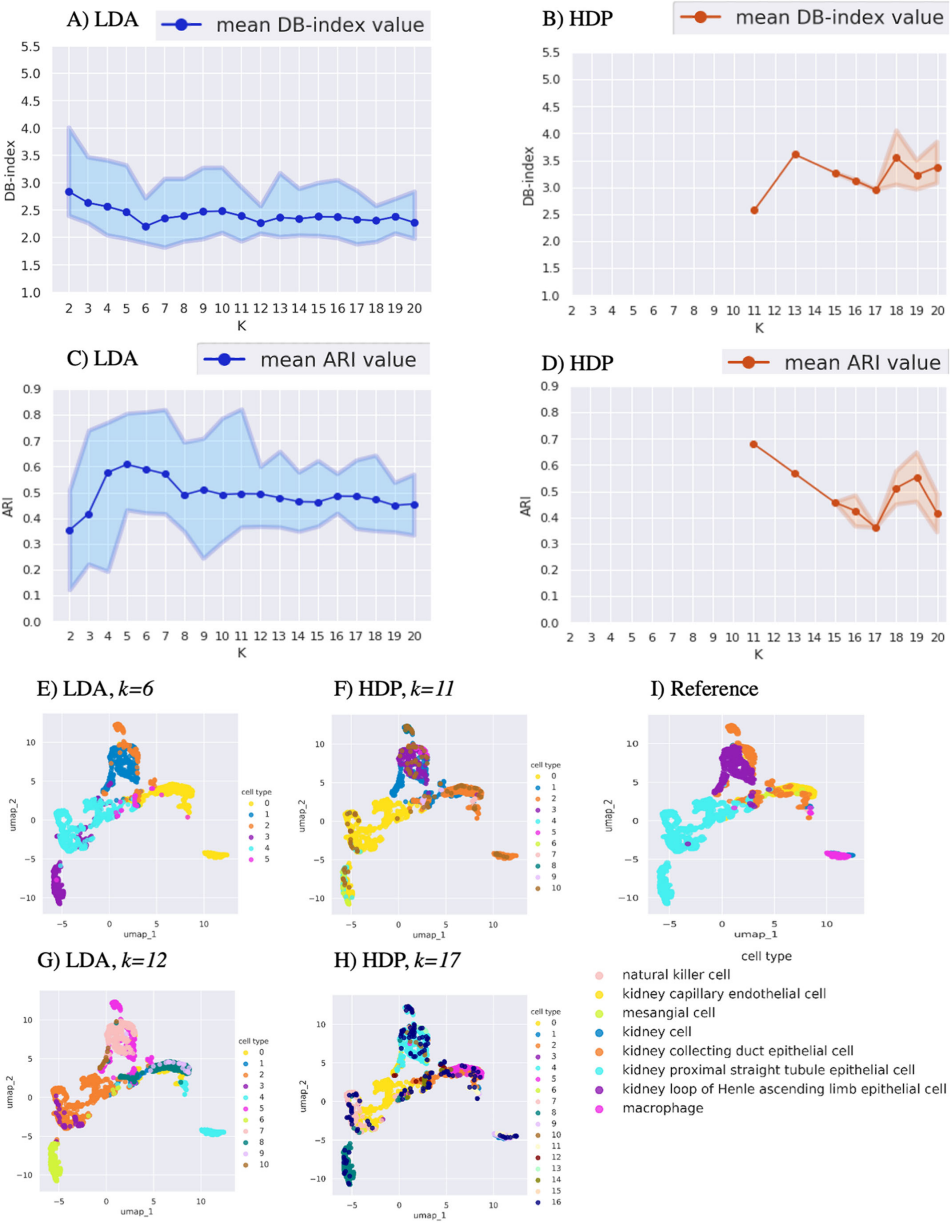
**Comparison of LDA and HDP clustering performance using mouse kidney cells (Schaum et al., 2018).** Intrinsic cluster quality measure defined by DB-index for (A) LDA and (B) HDP clustering. The *x*-axis shows the number of clusters *k*, and the *y*-axis indicates the DB-index values (lower indicates better clustering). Extrinsic cluster quality measure defined by ARI for (C) LDA and (D) HDP clustering. The *x*-axis shows the number of clusters *k*, and the *y*-axis indicates ARI (higher indicates better clustering). For (A-D) each run was repeated 20 times and the top, middle and bottom lines show the maximum, mean and minimum quality values, respectively. The UMAP plot of LDA clustering with (E) *k*=6 and (G) *k=*12. The UMAP plot of HDP clustering with (F) *k*=11 and (H) *k=*17. (I) The UMAP plot showing the reference clustering with the cell-type annotation from the original publication (Schaum et al., 2018).

In the intrinsic evaluation of the mouse kidney data (Schaum et al., 2018), LDA with cluster numbers *k*=6 and *k*=12 showed the minimum average DB-index values of 2.2 and 2.3, respectively (Fig. 2A), indicating the highest intrinsic cluster quality. The HDP clustering result partitioned the dataset into *k*=11 clusters with the lowest average DB-index value of 2.6 followed by *k*=17 with average DB-index value of 3.0 (Fig. 2B).

In the extrinsic comparison, LDA clustering with *k*=6 showed an average ARI value of 0.60, which was close to the highest average ARI value of 0.61 obtained with *k*=5 (Fig. 2C). The HDP clustering (*k*=11) had an average ARI value of 0.67 (Fig. 2D), suggesting that, in this dataset, the DB-index informed HDP with a higher cluster number (*k*=11) may be useful in order to achieve a more detailed cell state or subtype specific resolution than the DB-index informed LDA with *k*=6.

Visual inspection with the reference cell-type annotations indicated that LDA clustering with *k*=6 (best by DB-index) resolved the kidney limb epithelial cells, duct epithelial cells and partitioned the kidney tubule epithelial cells into two sub-clusters (Fig. 2E,I). However, it did not separate a cluster of immune cells (macrophages) from kidney cells, whereas LDA with *k*=12 did (Fig. 2G,I). The HDP clustering with *k*=11 gave similar results as LDA with *k*=12 (Fig. 2F), whereas HDP with *k*=17 added several apparently sporadic clusters (Fig. 2H). Considering the DB-index for the selection of an approximate cluster number, the HDP *k* value (*k*=11) had the lowest DB-index value (Fig. 2B) and highest ARI value (Fig. 2D), suggesting the utility of HDP in this dataset. Additionally, these results suggest that the HDP-based *k* value may be useful to guide the selection of the *k* value for LDA, when two LDA *k* values have similar DB-index.

### LDA and HDP clustering performance in mouse pancreatic cells

**Fig. 3.**
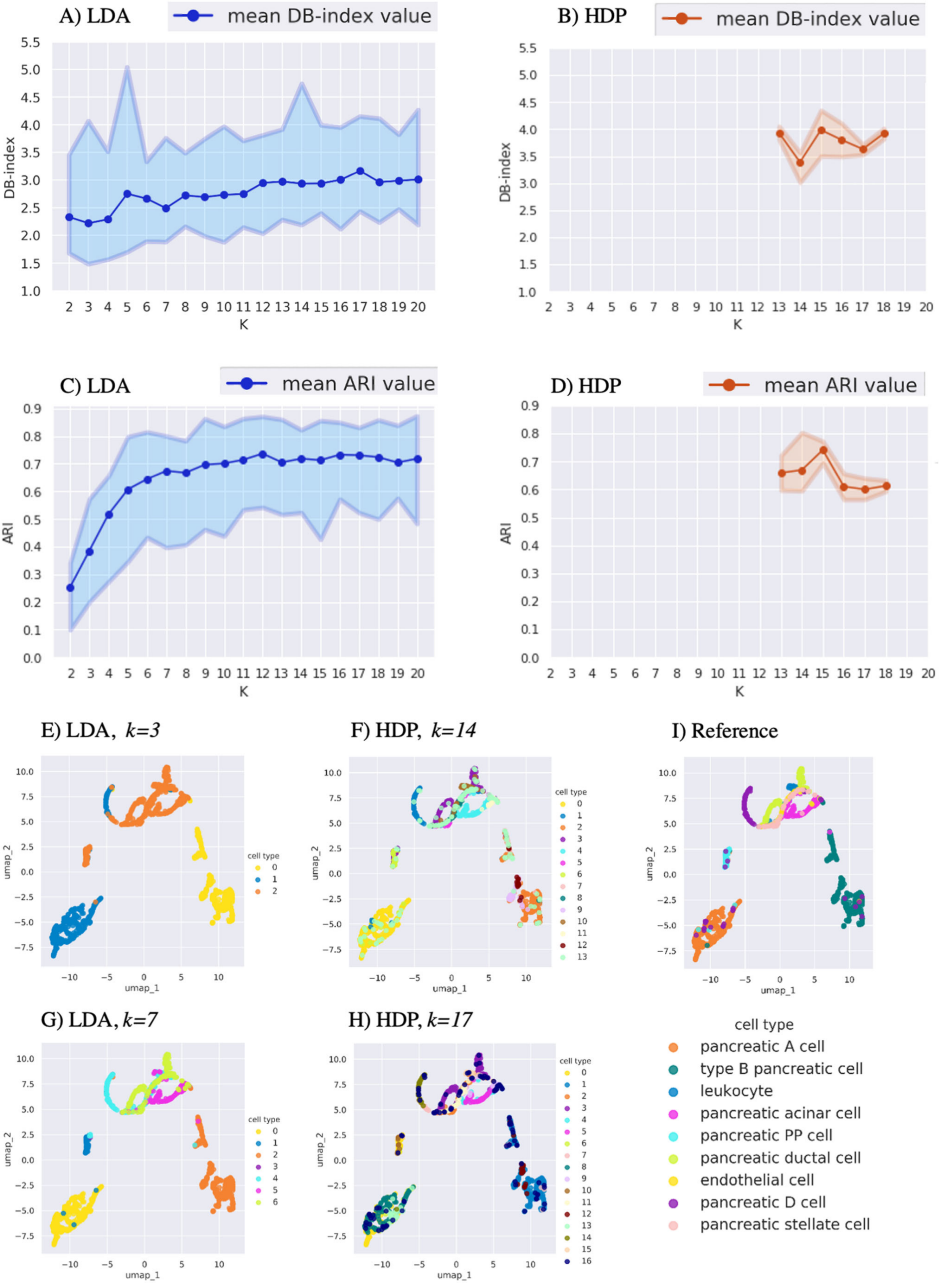
**Comparison of LDA and HDP clustering performance using mouse pancreatic cells (****Schaum et al., 2018****).** Intrinsic cluster quality measure defined by DB-index for (A) LDA and (B) HDP clustering. The *x* axis shows the number of clusters *k*, and the *y*-axis indicates the DB-index values (lower indicates better clustering). Extrinsic cluster quality measure defined by ARI for (C) LDA and (D) HDP clustering. The *x* axis shows the number of clusters *k*, and the *y*-axis indicates ARI (higher indicates better clustering). For (A-D) each run was repeated 20 times and the top, middle and bottom lines show the maximum, mean and minimum quality values, respectively. The UMAP plot of LDA clustering with (E) *k*=3 and (G) *k*=7. The UMAP plot of HDP clustering with (F) *k*=14 and (H) *k*=17. (I) The UMAP plot showing the reference clustering with the cell-type annotation from the original publication (Schaum et al., 2018).

We repeated the comparison of LDA and HDP using mouse pancreatic cells (Schaum et al., 2018). In the intrinsic evaluation, the LDA clustering with *k*=3 showed the lowest mean DB-index value of 2.3, and with increasing *k*, *k*=7 displayed a local minimum (DB-index=2.5) (Fig. 3A). Based on DB-index, HDP had worse performance compared to LDA, with *k*=14 showing the lowest average DB-index value of 3.4 (Fig. 3B), and *k*=17 showing the second lowest average DB-index value of 3.6.

In the extrinsic cluster quality evaluation, increasing the LDA cluster number from *k*=3 to *k*=7 increased ARI, but larger numbers of clusters did not affect the quality markedly, producing average ARI values in the range 0.67-0.72 (Fig. 3C). Similarly, the HDP clustering with *k*=14 gave an ARI value of 0.67 (Fig. 3D). The visual inspection with reference annotations suggested that LDA with *k*=7, but not with *k*=3, was able to resolve most of the cell subtypes present in the reference (Fig. 3E,G,I), whereas HDP with *k*=14 and *k*=17 resulted in additional cell subsets (Fig. 3F,H).

### Comparison of existing LDA clustering tools for scRNA-seq data

While the main aim of our study was to compare LDA and HDP for clustering scRNA-seq data, we also compared the Gensim implementation of LDA with two existing LDA implementations for scRNA-seq data, Celda (Wang et al., 2021) and DIMM-SC (Sun et al., 2018). Since the computational times with DIMM-SC extended to several weeks with the full datasets, we used the top 2000 most highly variable genes for this comparison (Figs S2 and S3). Especially with Gensim LDA and Celda, the best *k* values defined by the lowest DB-index values were generally in line with the highest average ARI values (Figs S2 and S3). On the other hand, while Gensim resulted in better (lower) mean DB-index values compared to the other two methods, Celda displayed higher extrinsic ARI values in the two datasets. This was also reflected in the UMAP visualization, where Celda resulted in coherent clustering of the cells (Figs S2 and S3). Overall, the Gensim LDA and DIMM-SC showed a wider range of variability in the cluster quality values than Celda for the repeated clustering runs (Figs S2 and S3).

### Comparison of LDA, HDP and the Seurat SNN clustering

The Bayesian Dirichlet process mixture models such as LDA and HDP are different from the clustering methods used in most of the existing state-of-art single-cell clustering tools, such as the widely used Seurat tool [20]. Seurat 4 clustering uses the graph-based shared nearest-neighbor (SNN) algorithm, where the resolution parameter (*r*) controls the resulting number of clusters. We compared LDA and HDP with Seurat 4 [20] using the top 2000 most highly variable genes (Figs S4-S7). For the immune cell dataset, the Seurat clustering resulted in the best intrinsic quality (lower DB-index) when the resolution r was below 0.1, resulting in *k*=5 or *k*=6 (Fig. S4). It also had the highest extrinsic cluster quality defined by ARI value of 0.62, while the highest average ARI values for LDA and HDP clustering were 0.54 (with *k*=8) and 0.61 (with *k*=7), respectively. For the kidney, pancreas and early pregnancy datasets (Figs S5-S7), Seurat, LDA and HDP clustering results had relatively similar average DB-index values for the different cluster numbers and resolution parameters, but Seurat resulted in slightly better ARI values compared to HDP and LDA.

## DISCUSSION

We have evaluated the clustering performance of Dirichlet process mixture models LDA and HDP on three scRNA-seq datasets using both intrinsic (Hassani and Seidl, 2017) and extrinsic (Amigó et al., 2009) cluster quality measures defined by DB-index (Davies and Bouldin, 1979) and ARI (Hubert and Arabie, 1985), respectively. For each dataset, we also selected two best cluster numbers (*k*) based on intrinsic DB-index for more detailed visual evaluation. The intrinsic cluster quality provides general information about how compact the data points are within the individual clusters and how well the different clusters are separated. Because intrinsic quality measures do not assess the biological relevance of the clusters, we also considered extrinsic cluster quality and using UMAPs visually compared the identified clusters to the clusters from the original publications. Overall, our study showed that the relative performance of LDA and HDP was dataset dependent and highlighted the importance of carefully assessing the number of clusters when analyzing scRNA-seq data.

The variation in DB-index and ARI values between repeated runs of LDA and HDP indicated that the clustering results varied for different runs of the same dataset. Therefore, average values over multiple runs were used to produce robust results for the comparative analysis. Further, we generally observed less variation in HDP runs compared to LDA runs, suggesting that HDP could provide more robust DB-index and ARI values.

Our comparison of LDA and HDP indicated that their performance was dataset dependent. In the immune cell dataset (Fig. 1), the DB-index informed HDP resulted in a more adequate clustering than the DB-index informed LDA when evaluated by both ARI and visual inspection with the original reference annotations. This provided evidence that at least in some cases HDP is a useful addition to the previously more widely employed LDA. For the other two datasets (Figs 2 and 3), HDP did not offer a clear advantage over LDA. In the kidney data, the DB-index informed HDP performed well judged by ARI, but in the visual inspection it did not provide conceivable advantage over the DB-index informed LDA (Fig. 2). In the pancreas data, HDP suggested higher numbers of clusters than LDA, while visual inspection suggested that these may inflate the clustering (Fig. 3).

For the purpose of our comparisons, the cluster annotations from the original studies were considered to provide adequate level of resolution and quality to be used as a reference in the extrinsic analysis and in the visual inspection of the best intrinsic DB-index defined cluster numbers. A more in-depth biological interrogation of the detailed clustering differences is outside of the scope of our comparison. The overall biological interpretation of the resulting cluster annotations typically demands integration with other methods, such as protein level studies and spatial analysis (Dey et al., 2017).

Recently, several single-cell specific implementations of LDA clustering have become available (Dey et al., 2017; duVerle et al., 2016; Sun et al., 2018; Wang et al., 2021), while the implementations of HDP clustering for scRNA-seq are limited. We extended our main HDP to LDA comparison to also include two scRNA-seq specific LDA implementations, Celda (Wang et al., 2021) and DIMM-SC (Sun et al., 2018). We observed that, based on intrinsic DB-index analysis, Gensim LDA performed better than Celda and DIMM-SC, whereas extrinsic ARI analysis supported the coherence of the Celda results. Celda also showed less variability between repeated runs than Gensim LDA and DIMM-SC.

The runtime and memory usage of both LDA and HDP for datasets with smaller numbers of cells (∼1000, ∼2000, ∼3000 cells) was practical for repeated analysis runs. However, for the large dataset (∼65,000 cells), the increased running time affected the practicality of their use. Additionally, the inference method used in a given LDA or HDP implementation also affects its run time. The Gensim implementations of LDA and HDP use the variational inference method (Blei and Jordan, 2006), which is easier to scale to high-dimensional data than sampling-based inference methods such as MCMC (Blei et al., 2017). The LDA tools Celda and DIMM-SC implement the expectation maximation algorithm for model parameter estimation and, in the context of this study, they appeared computationally adequate, especially, when focusing on the top 2000 most highly variable genes. Currently, BISCUIT (Prabhakaran et al., 2016), the single-cell specific implementation for HDP clustering, uses Gibb's sampling as the inference method. Gibbs sampling typically runs extensive iterations before it converges to the target posterior distribution, making it computationally expensive. Accordingly, a single run of BISCUIT using only the top 2000 most highly variable genes took more than three days, making the current implementation impractical for extensive comparisons. Therefore, further developments HDP specific to high dimensional scRNA-seq data could enhance the current computational challenge.

We also compared the performance of LDA and HDP with the graph-based SNN clustering implemented in the widely used Seurat 4 tool as a comparator method to inspect how the LDA and HDP clustering performed when evaluated with the existing state-of-art clustering method. HDP and LDA model-based clustering in general showed comparable results both in intrinsic and extrinsic evaluation measures when compared to Seurat based clustering. However, both LDA and HDP clustering resulted in markedly higher variation in the clustering results for the repeated runs compared to Seurat (Figs S4-S7).

Ideally, cluster analysis results from scRNA-seq data give meaningful approximations of biological cell types or states. In this regard, the nonparametric HDP clustering method, unlike the LDA, automatically generates the number of clusters without a predefined number of clusters (Limsettho et al., 2014; Teh et al., 2006). Thus, HDP avoids the additional analysis of different *k* values to select the optimal number of clusters. In addition to the direct use of HDP clusters, HDP could also be used for exploratory cluster analysis to visualize and explore the unknown cellular states from scRNA-seq data and to help guide the choice a suitable number of clusters as a starting point for more refined analysis. We observed that LDA performed more robustly in the data that had closely related cell types or states, and in these cases HDP may inflate the cluster number. On the other hand, the tendency of HDP to result in larger numbers of clusters than LDA may also open up the possibility of finding novel cell types or states, which is of high importance for both basic research as well as in the inference of disease specific conditions.

The study was limited to compare the LDA and HDP model-based clustering methods in only small to medium-sized single-cell RNA-seq data due to the very long execution time (several days) that it takes to run HDP models for large datasets. Additionally, the LDA and HDP models have multiple prior concentration parameters used as an input that can affect the clustering result. However, coherent parameter tuning for multiple parameters at the same time would have required extensive computational resources and was beyond the scope of this manuscript. Therefore, we limited our comparisons by fixing those concentration parameters to the default values.

In conclusion, our results support the previous reports that Dirichlet process based clustering models such as LDA and HDP are useful additions for single-cell data analysis in general (Bravo González-Blas et al., 2019; Kim et al., 2020; Sun et al., 2018) and that the non-parametric HDP model is a useful addition to the previously used LDA in particular.

## MATERIAL AND METHODS

### Sequencing data

We analyzed four publicly available scRNA-seq datasets, including artificially mixed human immune cells, tissue specific subsets of kidney and pancreas cells from *Tabula Muris* (Schaum et al., 2018) and human decidua/placenta (early pregnancy) data (Vento-Tormo et al., 2018) with high quality cell-type annotations. For the first dataset, we created an artificial mixture of human immune cells from seven publicly available scRNA-seq datasets from Gene Expression Omnibus (GEO): GSE75748, GSE81861, GSE44618, GSE96562, GSE85527, GSE96564 and GSE89232 (Table S1). The pre-processed datasets provided by the authors were downloaded from GEO together with their cell-type information, which was used as a reference in our clustering analysis. For the combined analysis, we converted raw counts and fragments per kilobase million (FPKM) to normalized transcripts per million (TPM) expression values, similarly as previously described (Pachter et al., 2011 preprint). The final artificial mixture contained expression profiles of 1153 human immune cells across 13,880 genes, including CD4+ memory cells, CD8+ memory cells, B cells, dendritic cells, fibroblasts, and lymphoblasts.

The mouse kidney and pancreas datasets were from the publicly available *Tabula Muris* study (Schaum et al., 2018). The unique molecular identifier (UMI) count matrix provided by the authors was downloaded from GEO with accession GSE109774. We selected the kidney (SMART-seq based) and pancreas (droplet-based) cells, including a total of 2782 and 1961 cells, respectively, with 23,433 genes for both datasets. The pre-processed UMI count data for human early pregnancy data (droplet-based) (Vento-Tormo et al., 2018) with 64,734 cells and 31,764 genes was downloaded from ArrayExpress with the accession number of E-MTAB-6701. We used the within cell UMI count library size normalization with scaling a factor of 10^6^ (Satija et al., 2015).

### LDA and HDP implementations

We used the python implementations for LDA and HDP originally designed for topic modelling from the ‘Gensim’ package. The benefit of using Gensim was that it has both LDA and HDP implemented in a single tool to ensure direct comparability. Additionally, the variational inference-based implementation of Gensim for LDA (Hoffman et al., 2010) and HDP (Wang et al., 2011) enabled scaling to high-dimensional datasets (Rehurek and Sojka, 2010). For the analysis, we used the normalized count data rounded to their nearest integer values in a ‘bag-of-words’ representation (Zhang et al., 2010) and default parameters (alpha=1 and eta=.01 for LDA; alpha=1, gamma=1 and eta=.01 for HDP). With LDA, the number of clusters *k* was varied from 2 to 20, whereas HDP does not have a predefined number of clusters. The soft/mixed cluster assignments were transformed to hard cluster assignments by assigning each cell to the cluster with the highest cluster membership probability. In order to have biologically interpretable clustering results, clusters with less than 15 cells were grouped as a separate single cluster. For the visualizations of the clustering results, we used the Uniform Manifold Approximation and Projection (UMAP) (McInnes et al., 2018).

In addition to the main LDA and HDP model comparison, we also compared the Gensim LDA implementation with two existing LDA implementations for scRNA-seq data, Celda (Wang et al., 2021) and DIMM-SC (Sun et al., 2018). Since the computational times with DIMM-SC extended to several weeks with the full datasets, for this additional analysis, we used only the top 2000 most highly variable genes. Again, default parameters were used, and the number of clusters *k* was varied from 2 to 20. Similarly, we attempted to compare the Gensim HDP implementation with the existing HDP implementation for single-cell RNA-seq data, BISCUIT (Prabhakaran et al., 2016). However, with BISCUIT, since the computational time for only a single cluster analysis run for pancreatic data, for example, with the top 2000 variable genes took more than 3 days, we excluded it from further analysis.

Finally, we compared the Gensim implementation of LDA and HDP with Seurat 4 SNN clustering (Hao et al., 2021). We used the 2000 most highly variable genes and the default parameters. For LDA clustering, we considered the number of clusters *k* ranging from 2 to 20 with 20 replicated runs for each *k*. The HDP clustering was also replicated 20 times with default resolution parameters. In the same way, we replicated the Seurat 4 SNN clustering 20 times with random seeding for multiple different resolution parameters ranging from 0.008 to 0.6.

### Measures of cluster quality

The cluster quality was assessed using both intrinsic and extrinsic cluster quality measures. The intrinsic cluster quality measures involve compactness and separation as a criterion for cluster evaluation (Hassani and Seidl, 2017), whereas the extrinsic cluster quality measures evaluate the overall clustering in comparison with a reference clustering (Amigó et al., 2009).

Davies-Bouldin index (DB-index) (Davies and Bouldin, 1979) was used as an intrinsic cluster quality metric, which uses the intra-cluster variance and inter-cluster separation to evaluate cluster quality. For a clustering result which partition data points into *k* clusters*,* the DB-index is given by:

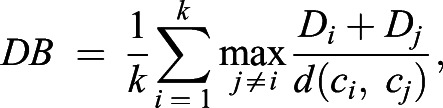
where *D_i_* is the average distance between all the data points in a given cluster *i* to their cluster center *c_i_* and *d(c_i_,c_j_)* is the distance between the *i^th^* and *j^th^* cluster centers. The smaller the DB-index, the better the compactness and separation of the clusters.

Calinski-Harabasz (CH-index) (Calinski and Harabasz, 1974) was also considered as another intrinsic cluster quality metric defined by the ratio of the overall between-cluster variance to overall within-cluster variance. The larger the CH-index, the higher the cluster quality.

Adjusted Rand Index (ARI) (Hubert and Arabie, 1985) was used as an extrinsic cluster quality measure, which extends the Rand index (*RI*) (Rand, 1971) of the similarity between two clusters to adjust for chance. Here, ARI was used as a measure of cluster accuracy by comparing the observed clustering with the reference clustering. Given a clustering result *X={X_1_,X_2_,…,X_k_}* and the reference clustering *Y={Y_1_,Y_2_,…Y_l_},* the ARI is given by:

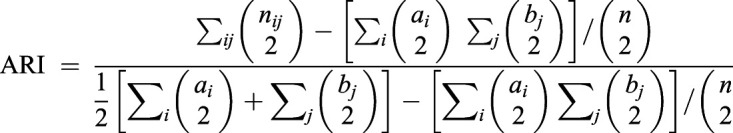
where *a_i_* is the number of data points in cluster *X_i_*, *b_j_* is the number of data points in cluster *Y_j_, n_ij_* is the number of overlapping data points in clusters *X_i_* and *Y_j_*, and *n* is the total number of data points. The higher the ARI value, the higher the agreement between the clustering results, with value of 1 being the maximum.

## Supplementary Material

Supplementary information
