# Supplementary material for: Dirichlet process mixture models for single-cell RNA-seq clustering
Source: Biol Open. 2022 Apr 4;11(4):bio059001. doi: 10.1242/bio.059001 (PMC9002799; doi:10.1242/bio.059001)
Supplement: Supplementary information [file biolopen-11-059001-s1.pdf]

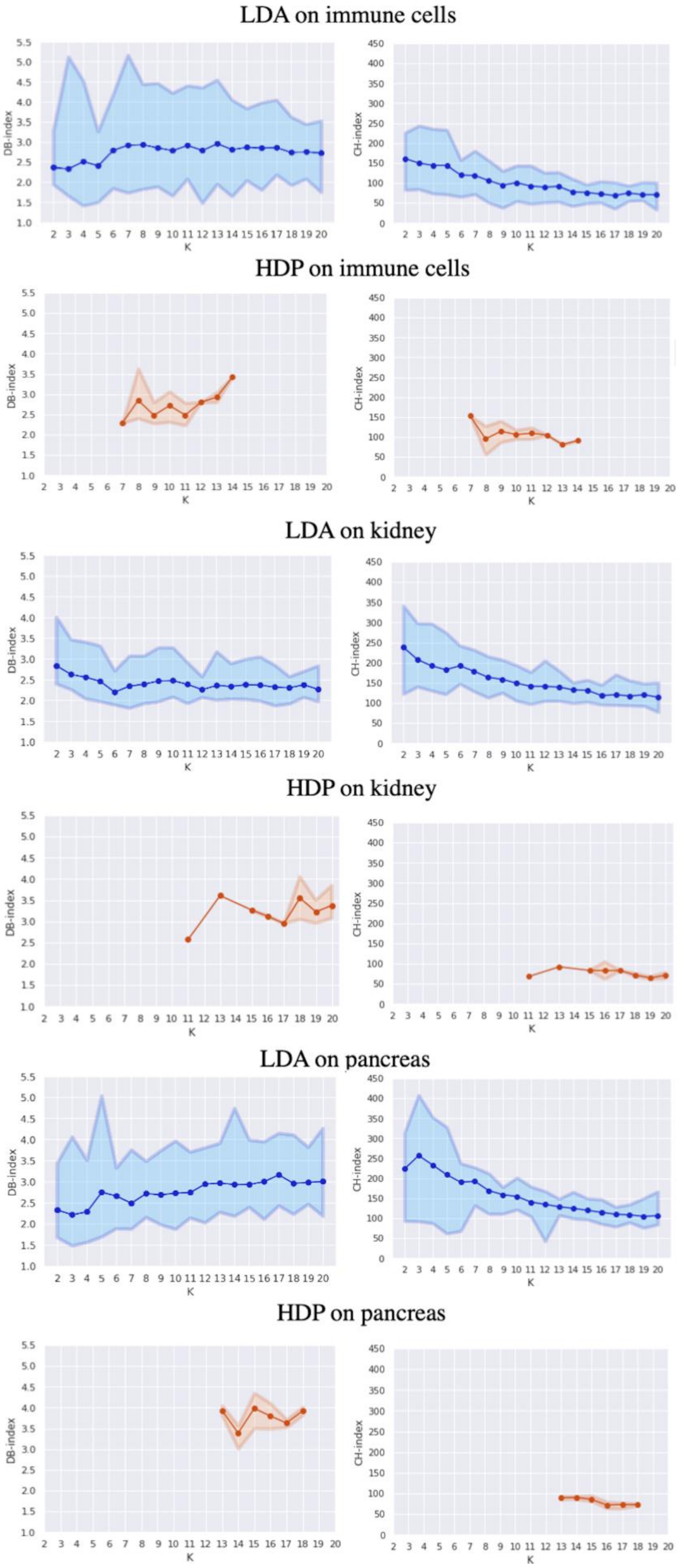

Biology Open • Supplementary information

Fig. S1. Intrinsic cluster quality measures defined by DB-index and CH-index for LDA and HDP clustering results on the artificially mixed human immune cell data and the mouse kidney and pancreatic cell datasets.

Biology Open • Supplementary information

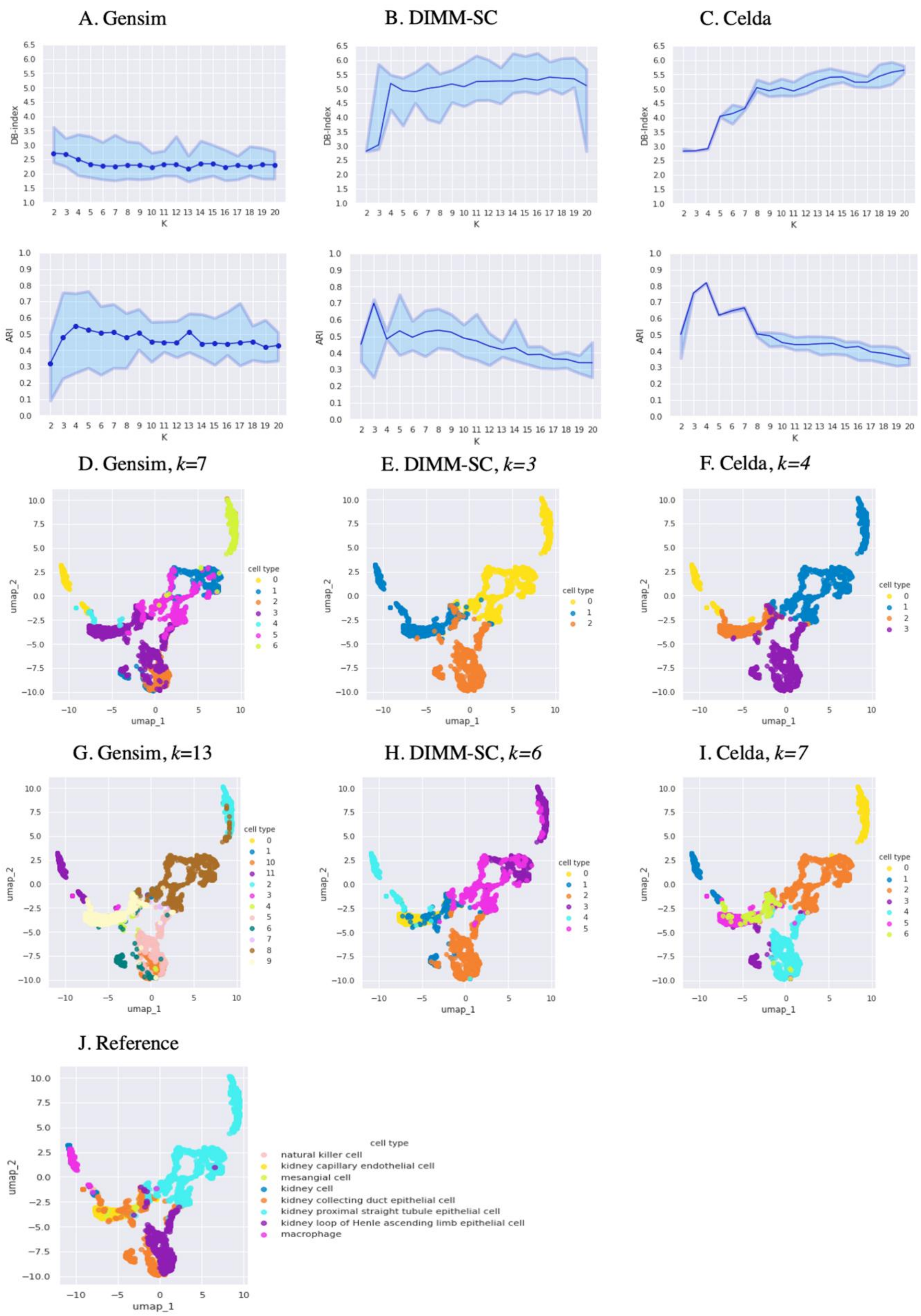
A. Gensim
B. DIMM-SC
C. Celda
DB-index
K
ARI
D. Gensim, k=7
E. DIMM-SC, k=3
F. Celda, k=4
G. Gensim, k=13
H. DIMM-SC, k=6
I. Celda, k=7
J. Reference
umap_1
umap_2
cell type
natural killer cell
kidney capillary endothelial cell
mesangial cell
kidney cell
kidney collecting duct epithelial cell
kidney proximal straight tubule epithelial cell
kidney loop of Henle ascending limb epithelial cell
macrophage

Fig. S2. Comparison of single-cell specific LDA clustering tools in the mouse kidney dataset: (A) Gensim LDA, (B) DIMM-SC, and (C) Celda. The intrinsic cluster quality measure was defined by Davies-Bouldin index (DB-index) and the extrinsic cluster quality measure by Adjusted Rand Index (ARI). The x-axis shows the number of clusters (k = *2-20)*, and the y-axis indicates the DB-index values (lower indicates better clustering) and ARI values (higher indicates better clustering). For (A-C) each run was repeated 20 times and the top, middle and bottom lines show the maximum, mean and minimum quality values, respectively. The UMAP plot of Gensim LDA clustering with (D) k = 7 and (G) k = 13. The UMAP plot of DIMM-SC clustering with (E) k = 3 and (H) k = 6. The UMAP plot of Celda clustering with (F) k = 4 and (I) k = 7. (J) The UMAP plot showing the reference clustering with the cell-type annotation from the original publications. The top 2000 most highly variable genes were used as input for the runs.

Biology Open • Supplementary information

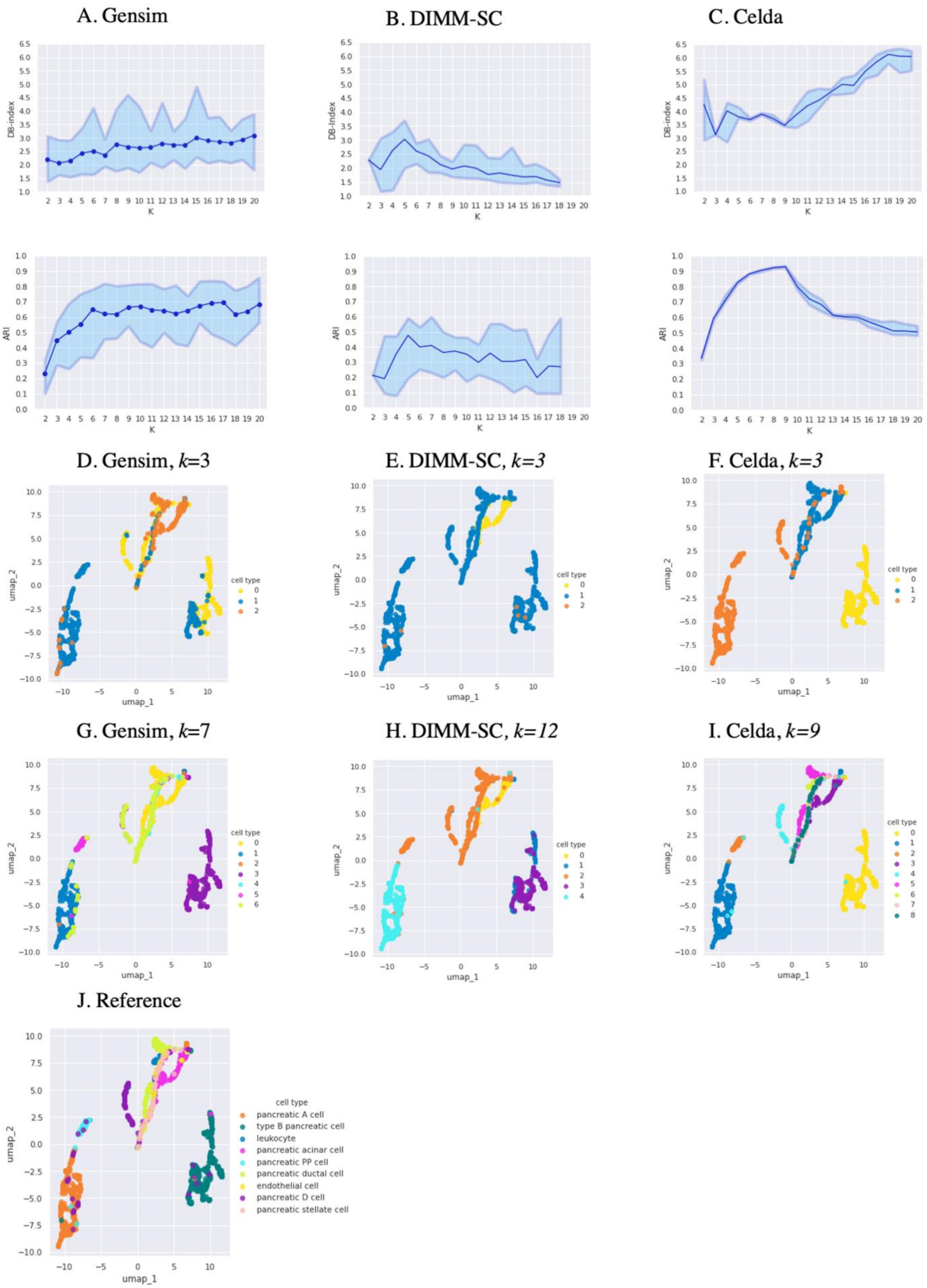
A. Gensim
B. DIMM-SC
C. Celda
DB-index
ARI
K
D. Gensim, k=3
E. DIMM-SC, k=3
F. Celda, k=3
G. Gensim, k=7
H. DIMM-SC, k=12
I. Celda, k=9
J. Reference
umap_1
umap_2
cell type
pancreatic A cell
type B pancreatic cell
leukocyte
pancreatic acinar cell
pancreatic PP cell
pancreatic ductal cell
endothelial cell
pancreatic D cell
pancreatic stellate cell

Fig. S3. Comparison of single-cell specific LDA clustering tools in the mouse pancreas dataset: (A) Gensim LDA, (B) DIMM-SC and (C) Celda. The intrinsic cluster quality measure was defined by Davies-Bouldin index (DB-index) and the extrinsic cluster quality measure by Adjusted Rand Index (ARI). The x-axis shows the number of clusters (*k=2-20)*, and the y-axis indicates the DB-index values (lower indicates better clustering) and ARI values (higher indicates better clustering). For (A-C) each run was repeated 20 times and the top, middle and bottom lines show the maximum, mean and minimum quality values, respectively. The UMAP plot of Gensim LDA clustering with (D) *k* = 3 and (G) *k* = 7. The UMAP plot of DIMM-SC clustering with (E) *k* = 3 and (H) *k* = 12 (clusters with less than 15 cells are collapsed). The UMAP plot of Celda clustering with (F) *k* = 3 and (I) *k* = 9. (J) The UMAP plot showing the reference clustering with the cell-type annotation from the original publications. The top 2000 most highly variable genes were used as input for the runs.

Biology Open • Supplementary information

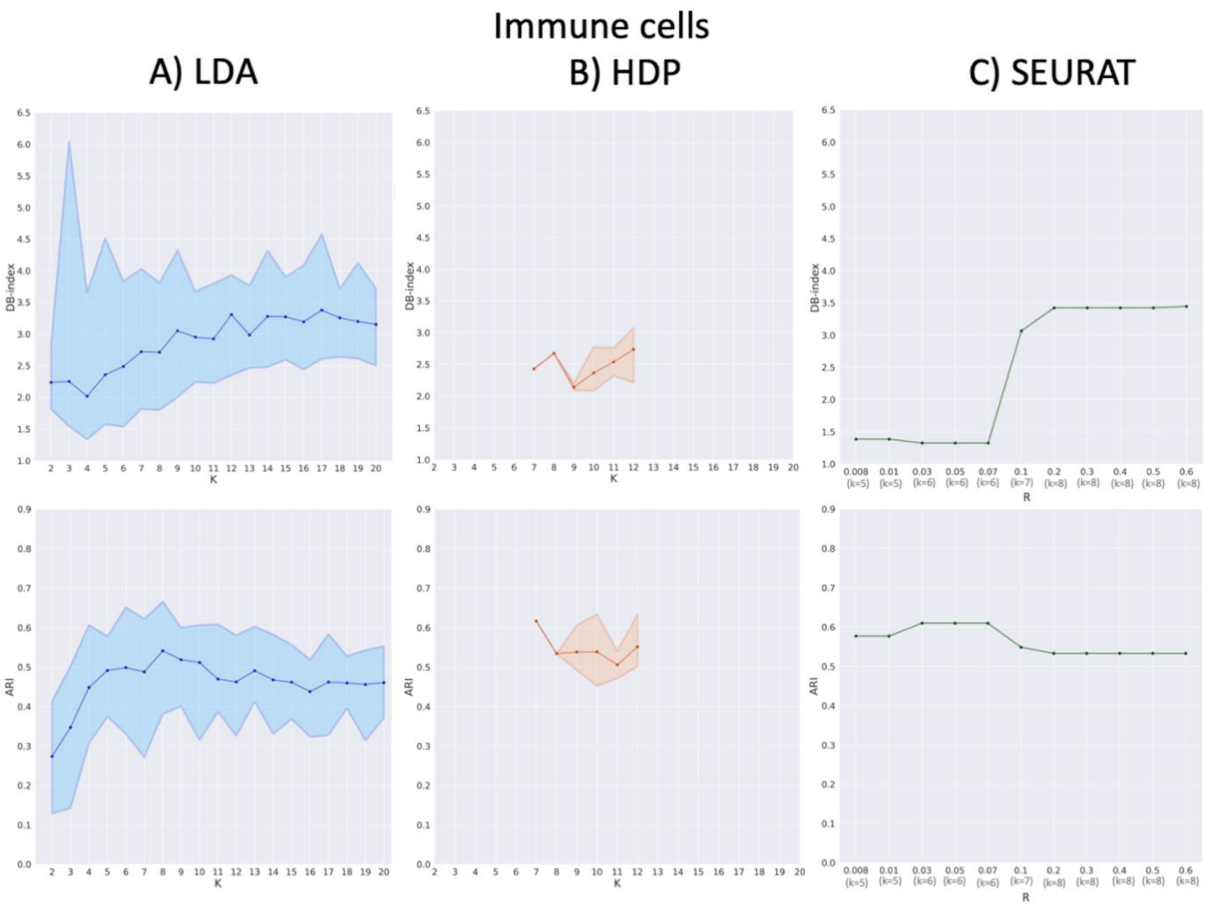

Fig. S4. Comparison of LDA, HDP and Seurat clustering based on intrinsic (Davies-Bouldin index, DB-index) and extrinsic (Adjusted Rand Index, ARI) cluster quality measures on immune cell data: (A) LDA, (B) HDP, and (C) Seurat SNN. The *x*-axis shows the number of clusters (*k=2-20)* for LDA and HDP and the resolution parameter *r* (from 0.008 to 0.6) for Seurat SNN. For Seurat, the average cluster numbers for the given resolution parameters are shown in brackets. The *y*-axis shows the maximum, mean and minimum values for DB-index (lower indicates better clustering) and ARI values (higher indicates better clustering) across 20 repeated runs.

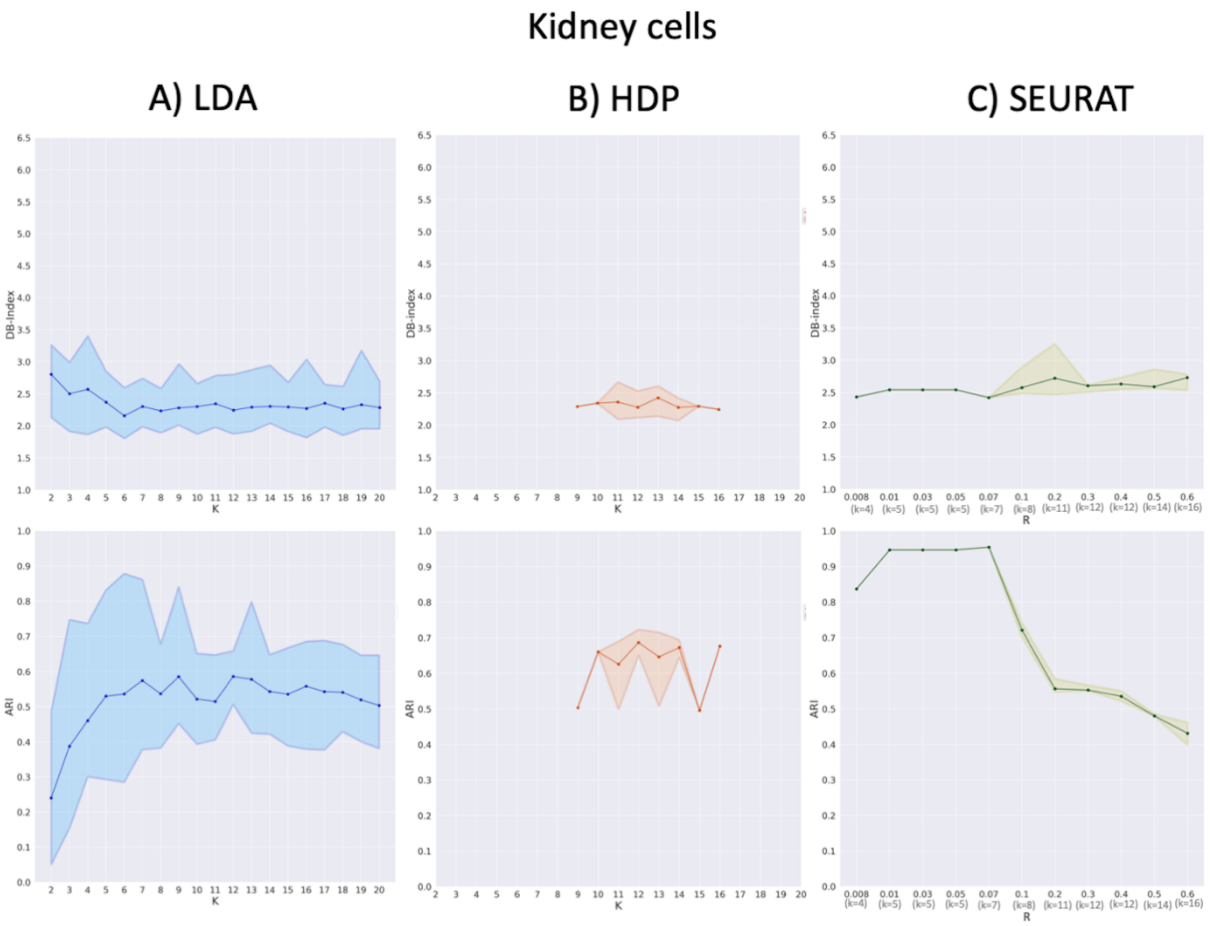

Fig. S5. Comparison of LDA, HDP and Seurat clustering based on intrinsic (Davies-Bouldin index, DB-index) and extrinsic (Adjusted Rand Index, ARI) cluster quality measures on mouse kidney cell data: (A) LDA, (B) HDP, and (C) Seurat SNN. The *x*-axis shows the number of clusters (k=2-20) for LDA and HDP and the resolution parameter *r* (from 0.008 to 0.6) for Seurat SNN. For Seurat, the average cluster numbers for the given resolution parameters are shown in brackets. The *y*-axis shows the maximum, mean and minimum values for DB-index (lower indicates better clustering) and ARI values (higher indicates better clustering) across 20 repeated runs.

Biology Open • Supplementary information

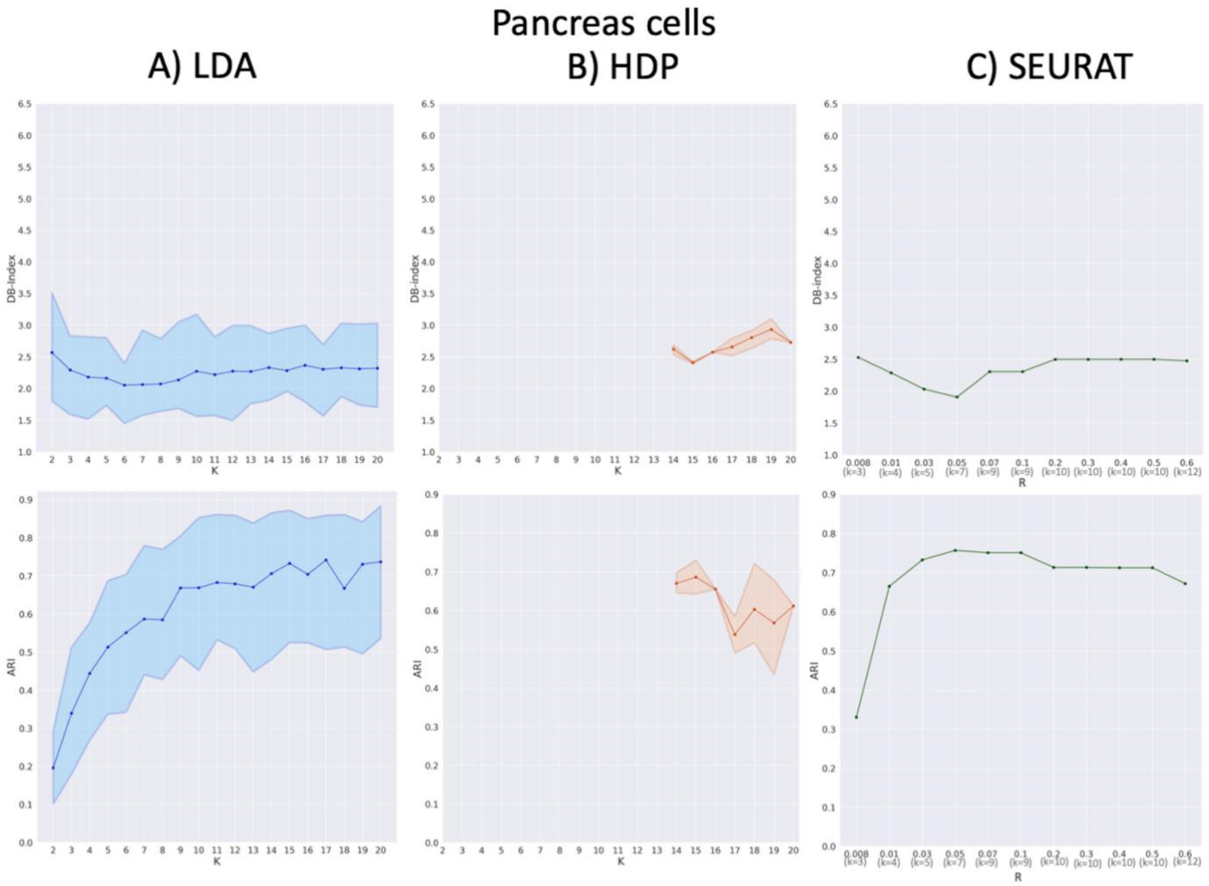

Fig. S6. Comparison of LDA, HDP and Seurat clustering based on intrinsic (Davies-Bouldin index, DB-index) and extrinsic (Adjusted Rand Index, ARI) cluster quality measures on mouse pancreas cell data: (A) LDA, (B) HDP, and (C) Seurat SNN. The *x*-axis shows the number of clusters (k=2-20) for LDA and HDP and the resolution parameter *r* (from 0.008 to 0.6) for Seurat SNN. For Seurat, the average cluster numbers for the given resolution parameters are shown in brackets. The *y*-axis shows the maximum, mean and minimum values for DB-index (lower indicates better clustering) and ARI values (higher indicates better clustering) across 20 repeated runs.

Biology Open • Supplementary information

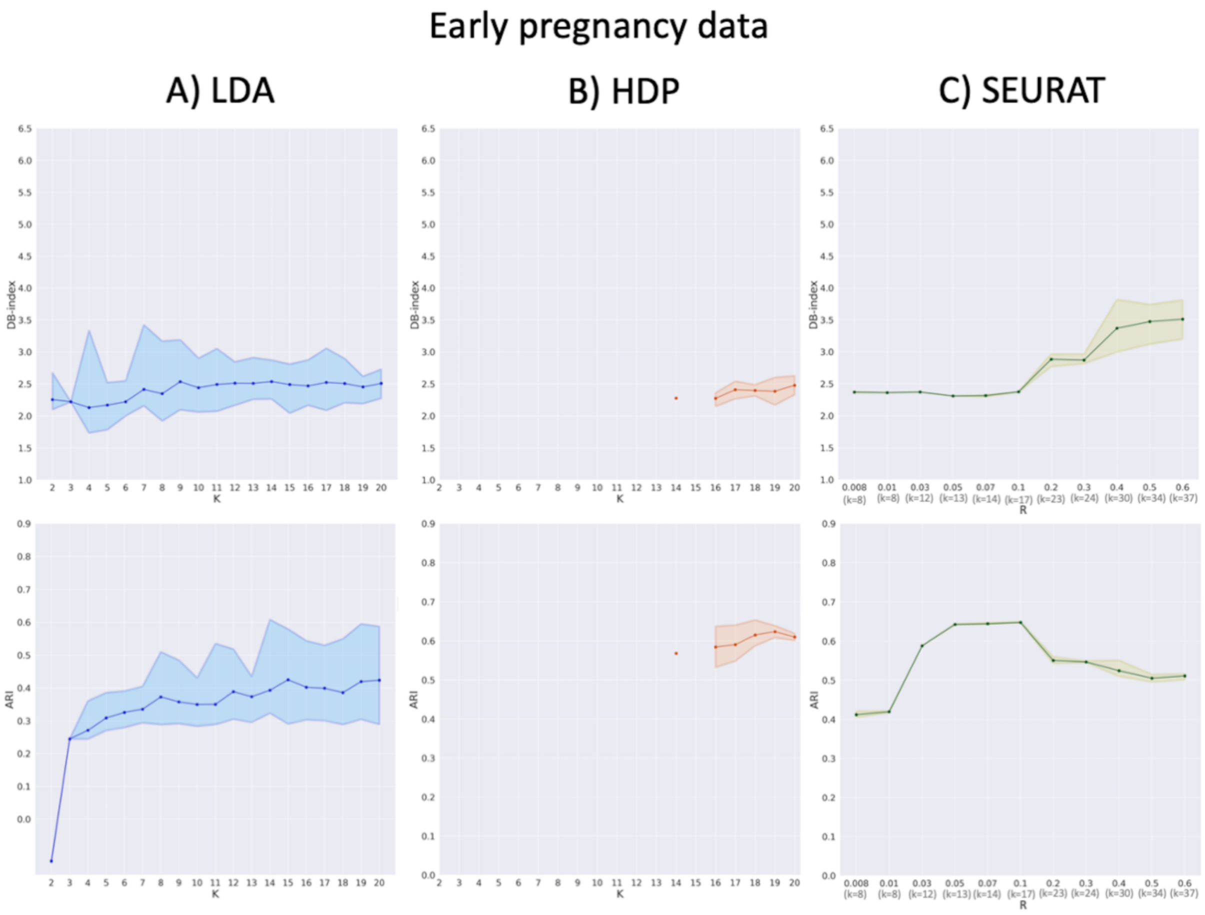

Fig. S7. Comparison of LDA, HDP and Seurat clustering based on intrinsic (Davies-Bouldin index, DB-index) and extrinsic (Adjusted Rand Index, ARI) cluster quality measures on human early pregnancy data: (A) LDA, (B) HDP, and (C) Seurat SNN. The *x*-axis shows the number of clusters (k=2-20) for LDA and HDP and the resolution parameter *r* (from 0.008 to 0.6) for Seurat SNN. For Seurat, the average cluster numbers for the given resolution parameters are shown in brackets. The *y*-axis shows the maximum, mean and minimum values for DB-index (lower indicates better clustering) and ARI values (higher indicates better clustering) across 20 repeated runs.

Biology Open • Supplementary information

Table S1. Artificial mixture of human immune cells.

Selected cells by Cell-type (n of cells)	GEO accession (n of cells)	Library preparation	Sequencing platform	Downloaded data format
Fibroblast (159)	GSE75748 (1810)	Fluidigm C1	Illumina HiSeq 2500	TPM
Lymphoblast (59)	GSE81861 (1220)	Fluidigm C1	Illumina HiSeq 2000	RPKM
B-cell (174)	GSE44618 (62)	SMART-seq 1	Illumina HiSeq 2000	RPKM
	GSE81861(1220)	Fluidigm C1	Illumina HiSeq 2000	RPKM
CD4+ memory T cell (393)	GSE96562 (149)	SMART-Seq 1	Illumina HiScanSQ	Raw count data
	GSE96568 (246)	SMART-Seq 1	Illumina HiSeq 2500	Raw count data
CD8+ memory T cell (263)	GSE85527 (219)	Nextera XT DNA Library Preparation Kit (Illumina)	Illumina HiSeq 2500	Raw count data
	GSE96564 (45)	SMART-Seq 1	Illumina HiSeq 2500	Raw count data
Conventional dendritic cell (105)	GSE89232 (957)	SMART-Seq 2	Illumina HiSeq 2500	TPM

Biology Open • Supplementary information

Table S2. Running time and memory usage for Gensim LDA and HDP clustering.

	Artificially mixed immune dataset		Pancreas, Tabula muris		Kidney, Tabula muris		Decidua/placenta	
	# genes	# cells	# genes	# cells	# genes	# cells	# genes	# cells
	13,000	1,153	23,000	1,961	23,000	2,782	23,000	64,734
LDA	1.7 min/ 2.6 GB		2.8 min/ 4.2 GB		2.3 min/ 6.0 GB		1.35 hrs/208 GB	
HDP	5.7 min/ 2.7 GB		15.2 min/ 4.3 GB		28.1 min/ 6.1 GB		4 days/ 208 GB	

Biology Open • Supplementary information
